# Daycare attendance and risk of childhood acute lymphoblastic leukaemia

**DOI:** 10.1038/sj.bjc.6600274

**Published:** 2002-05-06

**Authors:** X Ma, P A Buffler, S Selvin, K K Matthay, J K Wiencke, J L Wiemels, P Reynolds

**Affiliations:** Division of Public Health Biology and Epidemiology, University of California, Berkeley, California, CA 94720-7360, USA; Department of Pediatrics Oncology, University of California, San Francisco, California, CA 94143, USA; Department of Epidemiology and Biostatistics, University of California, San Francisco, California, CA 94143, USA; Environmental Health Investigations Branch, California Department of Health Services, Oakland, California, CA 94612, USA

**Keywords:** childhood leukaemia, daycare, delayed exposure

## Abstract

The relationship between daycare/preschool (‘daycare’) attendance and the risk of acute lymphoblastic leukaemia was evaluated in the Northern California Childhood Leukaemia Study. Incident cases (age 1–14 years) were rapidly ascertained during 1995–1999. Population-based controls were randomly selected from the California birth registry, individually matched on date of birth, gender, race, Hispanicity, and residence, resulting in a total of 140 case–controls pairs. Fewer cases (*n*=92, 66%) attended daycare than controls (*n*=103, 74%). Children who had more total child–hours had a significantly reduced risk of ALL. The odds ratio associated with each thousand child–hours was 0.991 (95% confidence interval (CI): 0.984–0.999), which means that a child with 50 thousand child–hours (who may have, for example, attended a daycare with 15 other children, 25 h per week, for a total duration of 30.65 months) would have an odds ratio of (0.991)^50^=0.64 (95% CI: 0.45, 0.95), compared to children who never attended daycare. Besides, controls started daycare at a younger age, attended daycare for longer duration, remained in daycare for more hours, and were exposed to more children at each daycare. These findings support the hypothesis that delayed exposure to common infections plays an important role in the aetiology of childhood acute lymphoblastic leukaemia, and suggest that extensive contact with other children in a daycare setting is associated with a reduced risk of acute lymphoblastic leukaemia.

*British Journal of Cancer* (2002) **86**, 1419–1424. DOI: 10.1038/sj/bjc/6600274 www.bjcancer.com

© 2002 Cancer Research UK

## 

Despite decades of research, the aetiology of childhood leukaemia remains obscure. Greaves hypothesised that delayed exposure to common infections leads to an increased risk of childhood leukaemia, especially common pre-B acute lymphoblastic leukaemia (ALL) (cALL), which has a maximum incidence in 2–5 year olds (Greaves, 1988, 1997). The essence of Greaves' hypothesis is that two separate genetic events may be responsible for the development of cALL; the first event occurs spontaneously pre- or peri-natally during the expansion of B-cell precursors; the second occurs in the same mutant clone following antigenic challenge early in life. A delay in a child's exposure to common childhood infections may result in an improperly modulated immune system and subsequent risk of aberrantly high levels of lymphoblastic cell proliferation following the barrage of infections when the child enters daycare or preschool. Intrigued by a leukaemia cluster in Seascale, UK, Kinlen suggested that childhood leukaemia might result from a rare response to a common (but identified) infection. Increased risks would occur when populations from different geographic areas were mixed so that infected and susceptible individuals had an increased level of contact (Kinlen, 1988, 1998). The hypotheses by both Greaves and Kinlen are consistent with the proposition that leukaemia may be a rare response to a common infection or infections.

In developed countries, most of a child's exposure to common infections is from contact with other children. It has been well documented that daycare attendance increases the risk of infections, which seems to increase with the number of children in a group (Istre *et al*, 1985; Ferson, 1993; Nystad *et al*, 1999). Alexander suggested that daycare of the index child and of the siblings is probably the best available proxy measure of exposure to infections (Alexander *et al*, 1993). To date, daycare/preschool (referred to as ‘daycare’ from here on) attendance, along with other variables such as birth order and number of siblings, has been used as an indicator of exposure to common infections in several studies (Petridou *et al*, 1993, 1997; Dockerty *et al*, 1999; Infante-Rivard *et al*, 2000; Neglia *et al*, 2000; Rosenbaum *et al*, 2000). However, the exposure assessment was crude in these studies and the results are inconsistent. The Northern California Childhood Leukaemia Study (NCCLS), which collected detailed data on contact with other children in a daycare setting, offered an opportunity to critically explore the quantitative relationship between daycare attendance and the risk of ALL.

## METHODS

### Study population

The NCCLS is currently ongoing, and is composed of two phases. Phase I consists of cases diagnosed between January 1, 1995 and November 30, 1999, and Phase II is planned to include cases diagnosed between December 1, 1999 and December 31, 2003. The cases in this analysis are from Phase I. Incident cases of newly diagnosed childhood leukaemia (age 0–14 years) were prospectively ascertained (usually within 24 h after diagnosis) from major clinical centres. Although case ascertainment was hospital-based, a comparison with cases ascertained by the statewide California Cancer Registry (1997–1999) shows that the NCCLS protocol successfully identified 88% of all newly diagnosed childhood leukemia cases in the San Francisco–Oakland Metropolitan Statistical Area. Controls were randomly selected from the statewide birth certificate files maintained by the California Department of Health Services and 1:1 matched to cases on date of birth, gender, mother's race (white, black, or other), Hispanicity (either parent is Hispanic), and mother's county of residence at the time of child's birth. These controls were then traced by using commercially available searching tools. For cases not born in California (less than 10% of all cases), county of residence at diagnosis was used for the matching. For each case, the search continued until an eligible control subject consented to participate in the study.

To be eligible, each case or control had to (1) reside in the study area; (2) be under 15 years of age; (3) have at least one parent/guardian who spoke English or Spanish; and (4) have no previous history of any malignancy. Of a total of 273 eligible Phase I cases, 229 (83%) consented to participate. This analysis is based on 183 cases for whom a matched control subject was available at the time of data analyses. The characteristics (age, gender, race, ethnicity, subtype, maternal education, household income, and daycare attendance) of these 183 cases were comparable to the cases who were not included in this analysis. In order to enrol the 183 controls, a total of 475 searches were conducted, from which 355 (75%) potential controls were successfully located. Of the 355 potential controls, 279 (79%) were eligible, of which 183 (66%) consented to participate. The actual number of searches per case ranged from 1 to 15. Of the 183 controls, 121 (66%) were the first choices, i.e. the first eligible controls contacted. The study protocol was approved by the Internal Review Board of all collaborating institutions, and written informed consents were obtained for all participating subjects. Epidemiologic data were obtained by a self-administered questionnaire and a subsequent in-home personal interview. Of the 183 cases, 148 had a diagnosis of ALL. Since infant ALL (diagnosed at a age of less than 12 months) is often considered to have a distinctive aetiology, eight infant ALL cases were excluded, resulting in a total of 140 ALL case–control pairs for the daycare analysis.

### Data collection

Data on daycare attendance were first collected by a self-administered questionnaire and then verified during an in-home personal interview. The interviews were conducted with the primary care givers, usually the biological mother (97%) or biological father (2.5%). All data for daycare attendance were censored on the date of diagnosis (corresponding dates for controls) or 6 years of age, whichever was earlier.

For this analysis, the following variables were included or constructed: (1) attendance at daycare (yes/no); (2) age when first started daycare (in months); (3) duration of stay at all daycare facilities (in months), regardless of hours attended; (4) mean hours per week attending daycare facilities; (5) mean number of children exposed to at each daycare facility; (6) total number of children ever exposed to in a daycare setting; and (7) total child–hours, which is an overall summary of daycare attendance. If a child only attended one daycare facility, then the mean number of children would be the same as the total number of children. Child–hours at each daycare is calculated as follows: number of months attending a daycare × mean hours per week at this daycare × number of other children exposed to at this daycare multiplied by 4.35 (i.e. number of weeks per month). The child–hours at each daycare were added to obtain the total child–hours for each child. For children who never attended daycare, an age of 72 months was assigned as the age when first started daycare, and a value of 0 was assigned as duration of stay, mean hours per week, mean number of children, total number of children, and total child–hours.

Data on the number of other children in household before the index child went to first grade were also collected. This was used as a potential indicator of exposure to infectious agents by contact with other children in the same household. In the United States, children usually start first grade when they are around 6 years of age.

### Statistical analysis

Pearson's Chi-square test was used to compare the distributions of demographic, socio-economic, and birth characteristics between cases and controls. The matched design of the NCCLS dictates a matched analysis. Paired *t*-tests were used to compare daycare attendance measures between cases and controls, one at a time. A quantile–quantile plot, which is an important graphic technique for comparing shapes of distributions (Hoaglin *et al*, 1985), was used to illustrate the difference in total child–hours between cases and controls. Nonparametric Wilcoxon signed-rank test and Kolmogorov–Smirnov test, which are robust and free from assumption of the distribution of data, were also conducted. Hotelling's multivariate T^2^ test provided a single assessment of the overall difference between cases and controls, measured in terms of the six variables. Multivariate conditional logistic regression was employed to estimate odds ratios and 95% confidence intervals, adjusting particularly for annual household income.

## RESULTS

Data on demographic, socio-economic, and birth characteristics of cases and controls were obtained from the in-home personal interviews, during which a respondent, usually the biological mother, was asked to indicate the race and ethnicity of the case or control child (Table 1

**Table 1:**
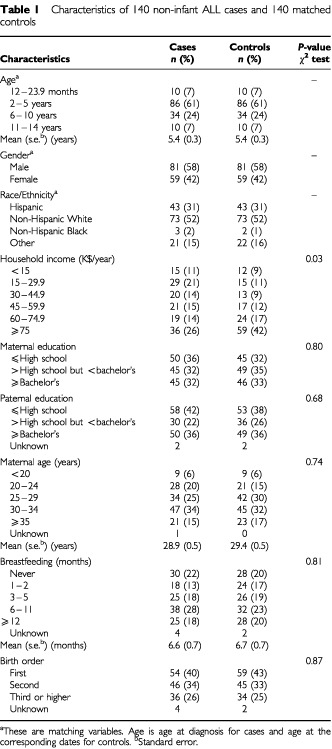
Characteristics of 140 non-infant ALL cases and 140 matched controls

). As expected, there was only a minor difference on race and Hispanicity between cases and controls. The distribution of cases and controls were similar with respect to maternal education, maternal age, duration of breastfeeding, and birth order. Controls appeared to have higher annual household income (*P* value=0.03).

Among the 140 pairs, 92 (65.7%) of the cases and 103 (73.6%) of the controls attended at least one daycare facility. Compared to the cases, controls started daycare at younger ages, stayed at daycare for longer, were exposed to more children in the daycare setting, and acquired more child–hours (Table 2

**Table 2:**
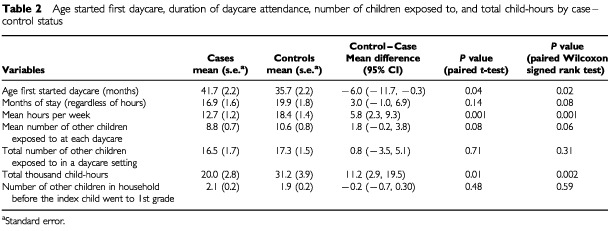
Age started first daycare, duration of daycare attendance, number of children exposed to, and total child-hours by case–control status

). Generally, significance levels from Wilcoxon signed-rank tests, which do not assume normal distributions, were smaller than those from paired *t*-tests. The mean number of other children in the household before the index child went to first grade was slightly higher in cases than in controls. Although not all the tests were statistically significant, there was a consistent pattern.

**Figure 1:**
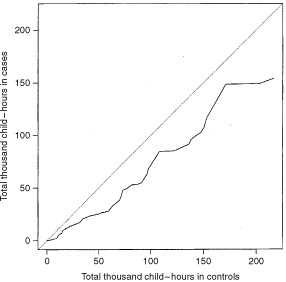
Quantile–quantile plot of total thousand child–hours.

The quantile–quantile plot (cases *vs* controls) clearly showed that cases had fewer total child–hours than the controls (Figure 1

). If there were no difference in the distribution of total child–hours between cases and controls, the points would have randomly deviated from the dotted reference line, as opposed to clustering in one side of the reference line. A nonparametric Kolmogorov–Smirnov test was conducted to compare the cumulative density functions of total child–hours between cases and controls, which produced a *P* value of 0.094. As expected, the other five daycare attendance variables in Table 2 followed the same pattern, since these daycare measures are all related.

Hotelling's multivariate T^2^ test was used to assess the overall difference between cases and controls, in terms of age started first daycare, duration of daycare attendance, mean hours per week, mean number of children at each daycare, total number of children at all daycares, and total child–hours. The T^2^ statistic was 3.74 with a corresponding *P* value of 0.003, which again indicates that one or more of the variables reflecting daycare exposure are associated with the risk of ALL.

The results presented above are not adjusted for household income. Since income is one of many measures of socio-economic status, and socio-economic status is possibly related to daycare attendance, household income was considered an important contributor in the conditional logistic regression analysis. Each unit of mean hours per week and total thousand child–hours is significantly associated with the risk of ALL, with and without controlling for household income (Table 3

**Table 3:**
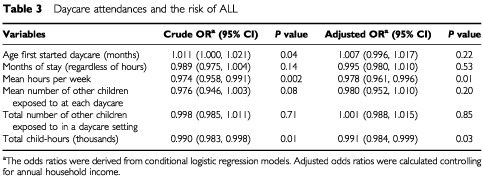
Daycare attendances and the risk of ALL

). The association between age first started daycare and ALL was statistically significant only when household income was not adjusted for. A decreased risk was also observed for children who stayed for longer, or exposed to more children, although the associations did not reach statistical significance.

**Figure 2:**
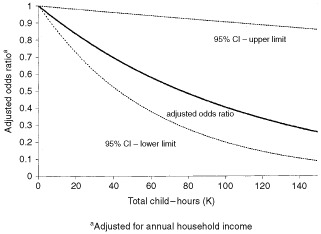
Total child–hours and the risk of childhood ALL.

The odds ratios displayed in Table 3 measure the change in risk associated with each unit of change of the independent variables. For example, each thousand child–hours is associated with an odds ratio of 0.991, which means that a child who has 50 thousand child–hours (who may have, for example, attended a daycare with 15 other children, 25 h per week, for a total duration of 30.65 months) would have an odds ratio of (0.991)^50^=0.64, compared to children who never attended daycare (Figure 2

).

A logistic regression model allows the simultaneous assessment of the impact of multiple daycare attendance variables on the difference in risk between cases and controls. In addition, the relative roles of each variable can be described and the confounding influence of income measured. The results from a conditional logistic analysis, based on a purely additive model employing the daycare attendance variables directly as measured, are presented in Table 4

**Table 4:**
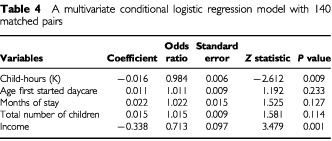
A multivariate conditional logistic regression model with 140 matched pairs

. Both mean hours per week and mean number of children exposed to at each daycare are excluded because they are highly correlated with other important variables included in the model. The exposure measured by total child–hours is the dominant predictor of case–control status (*z*=−2.612; *P* value=0.009). The other three exposure variables add little additional independent information to the evaluation of association between daycare attendance and the risk of ALL. Income is an important predictor of difference in risk between cases and controls, but is only a slight confounder of the daycare attendance variables. For example, the coefficient associated with total–child hours is −0.016 when income is included in the model, and is slightly smaller (−0.013) when income is not included, changing the *P* value from 0.009 to 0.022. The other daycare variables are similarly influenced by income, each showing a slightly less but still unimportant association with case–control status.

The protective effect of daycare attendance sustained in both Hispanic and White non-Hispanic subgroups. Separate analyses were conducted for 86 ALL cases who were diagnosed at 2–5 years old and are likely to be cases of cALL. The results derived from the analyses of these 86 case and birth certificate control pairs were not much different. The odds ratio adjusted for income is 0.991, which is the same as the odds ratio obtained from the analysis of the 140 case–control pairs. The odds ratio adjusted for age first started daycare, months of stay, total number of children, and income simultaneously is 0.985, which is very similar to the result based on the 140 case–control pairs (Table 4).

Daycare attendance did not appear to be associated with the risk of AML (OR=1.000, 95% CI: 0.984–1.016). However, due to the very limited number of AML case–control pairs available for analysis (*n*=35), the result should be interpreted with caution.

Excluding cases who were born out of the state of California (less than 10% of all the cases) did not alter any of the results.

## DISCUSSION

Our observation that extensive contact with other children in a daycare setting is associated with a reduced risk of childhood ALL provides strong indirect support to the hypothesis that delayed exposure to common infections is associated with the risk of ALL. Although the magnitude of the association we observed between daycare attendance and the risk of ALL is not large, it is consistent, not likely to be confounded by income, and statistically significant. Daycare attendance only reflects exposure to common infections, and is certainly, at best, an indirect measure. There are also background exposures that a child may experience through sources other than daycare attendance, such as contact with other children who live in the same household or in the public settings (hospitals, parks, shopping malls, etc.), and infectious agents brought home by parents.

Household income may reflect to some extent possible differences in socio-economic status not controlled for by the matching on race and Hispanicity. No income data were available for the nonparticipating controls. However, we were able to obtain information on maternal education, a commonly used marker for socio-economic status, from the birth certificates of all potential controls, whether or not they participated. The eligible first-choice controls (i.e. ideal controls), regardless of participation, have very similar maternal education levels to the controls who actually participated in the study (i.e. actual controls).

Three previous studies also reported that a larger proportion of controls attended daycare than cases (Petridou *et al*, 1993, 1997; Infante-Rivard *et al*, 2000). In a Greek study, childhood leukaemia cases in metropolitan Athens were less likely than controls to have attended daycare (OR=0.7, 95% CI: 0.4–1.1), especially for attendance of at least 3 months prior to the age of 2 years (OR=0.3, 95% CI: 0.1–0.9) (Petridou *et al*, 1993). Another study in Greece reported a similar association comparing the proportion of children who ever attended any daycare among the cases and controls, but the association was not statistically significant (OR=0.83, 95% CI 0.51–1.37) (Petridou *et al*, 1997). A significantly protective effect of daycare attendance was observed in a population-based Canadian study with ALL cases aged 0–9 years. Compared with children who never attended daycare, the odds ratios for children who started daycare at ⩽2 years old and >2 years old were 0.49 (95% CI: 0.31–0.77) and 0.67 (0.45–1.01), respectively (Infante-Rivard *et al*, 2000). No detailed information on daycare attendance, however, was reported in these three studies, which precluded directly comparing our study with theirs.

Dockerty *et al* (1999) found no significant association between attendance at particular types of preschool groups and ALL risk in New Zealand (OR=0.67, 95% CI 0.40–1.12). The relationship between daycare attendance and ALL was also evaluated in a recent study, which recruited cases from many Children's Cancer Group (CCG) member institutions across the United States (Neglia *et al*, 2000). Neither attendance at daycare, age first started daycare, or time enrolled at daycare, was associated with risk of overall ALL or cALL in the CCG study. Several explanations exist for the discrepancy between the CCG study and the NCCLS. Compared to the large, predominantly White CCG study population that included cases diagnosed at hospitals in many different states, our study population is more geographically defined, ethnically diverse, better educated, and has a much higher household income. In the CCG study, less than 50% of control subjects attended any daycare, while 74% of controls subjects in our study did so. Another difference between the two studies is the selection of control subjects. The CCG study used the method of random-digit dialling, while the NCCLS selected controls from population-based statewide birth registry. The most noteworthy difference, however, is that daycare attendance data obtained in the CCG study were not as detailed or complete as the data obtained in the NCCLS. For example, neither number of children the index child was exposed to nor the hours the child spent at each daycare facility was collected in the CCG study. None of the five variables evaluated in the CCG study, including any daycare, age at start of daycare, time at daycare, daycare before age two, time at daycare before age 2 years, were found to be important predictors of the risk of ALL in our study. Rather, it was the more specific indicators of exposure such as total child–hours that appeared to be associated with a significantly modified risk in our study. Despite the large sample size, the CCG study did not provide adequate data to evaluate the relationship between daycare attendance and the risk of childhood ALL. In a more recent study that included ALL cases diagnosed at one of the four referral centres in western and central New York State, the odds ratio for children who stayed at home compared with those who attended daycare for greater than 36 months was 1.32 (95% CI: 0.70–2.52); the odds ratios for 1–18 and 19–36 months of daycare were 1.74 (95% CI: 0.89, 3.42) and 1.32 (95% CI: 0.64, 2.71), respectively (Rosenbaum *et al*, 2000). When the analysis was restricted to B-lineage ALL, the magnitude of association increased. The authors concluded, however, that their results did not support an association between daycare attendance and ALL, probably because the 95% confidence intervals of several odds ratios included 1.0. In fact, combining the data presented for B-lineage ALL in Table 5 in the publication by Rosenbaum *et al* (2000) produces a summary Mantel–Haenszel odds ratio of 1.63 (95% CI: 1.06–2.52), which indicates an overall association with the length of daycare attendance. Breaking continuous variables into categories, although conventional in epidemiological studies, does not use all the information available and certainly decreases statistical power under most circumstances (Selvin, 1996). Also, the choice of the number of categories and corresponding cut points can change the results, sometimes substantially. In addition to the difference in analytical strategy, our study and the study by Rosenbaum *et al* (2000) differed by participation (83% and 66% in cases and controls, respectively, *vs* 71% and 55% in cases and controls, respectively), as well as the method of data collection (in-home personal interview *vs* mailed self-administered questionnaire). In our study, the conditional logistic regression analysis indicates that total child–hours, which combine information from several measures of daycare attendance into a single value, effectively summarise the protective effect of attending daycare (Table 4).

A particular strength of our study is a detailed and timely exposure assessment. The NCCLS commenced in 1995. The investigators had the advantage of learning from and improving upon data collection instruments that were used and kindly made available by researchers from previous studies. Detailed data on when a subject started and stopped attending each daycare facility, number of hours on average he/she spent at the facility, as well as how many other children that he/she was exposed to at the facility, were obtained. As opposed to a telephone interview or a mailed questionnaire, all data on daycare attendance were collected first by a self-administered questionnaire and then verified at an in-home personal interview with the primary care giver of the subject, usually the biological mother. In addition, special efforts were made to rapidly ascertain incident cases and shorten the time between diagnosis (corresponding dates for controls) and exposure assessment. Bias pertaining to the difficulty of recalling should have been reduced. Another important feature of the NCCLS is the selection of population-based controls from statewide birth registry. Close collaboration with the California Department of Health Services resulted in an expeditious process of control selection. A series of methodological evaluations (manuscripts in preparation) indicate that the birth certificate controls are representative of the population base from which the cases arose.

A limitation of the study is the relatively small sample size, which reduces the power. On the other hand, the matched design and detailed daycare exposure measures should improve the precision of statistical analyses. The NCCLS is currently ongoing, and it will provide more information with regard to daycare attendance and the risk of ALL with the expansion of study population in the near future.

In summary, our study offered strong but indirect support to the hypothesis that delayed exposure to common infections plays an important role in the aetiology of childhood ALL. Daycare attendance appears to be associated with a reduced risk of childhood ALL. Our findings, if confirmed by future studies with detailed exposure assessment, will likely shed light on the immunological aetiology of childhood ALL and might eventually lead to preventative strategies.
